# Translation and validation of the European Health Literacy Survey Questionnaire (HLS-EU-Q47) into the Slovenian language

**DOI:** 10.1007/s11096-023-01610-z

**Published:** 2023-06-24

**Authors:** Nuša Japelj, Nejc Horvat

**Affiliations:** Department of Social Pharmacy, University of Ljubljana, Faculty of Pharmacy, Askerceva cesta 7, 1000 Ljubljana, Slovenia

**Keywords:** Health literacy, Questionnaire, Reliability, Translation, Validity

## Abstract

**Background:**

The European Health Literacy Questionnaire (HLS-EU-Q47) is a self-assessment tool for standardised measurement of health literacy.

**Aim:**

To translate HLS-EU-Q47 into the Slovenian language and to investigate its reliability and validity in Slovenia.

**Method:**

HLS-EU-Q47 was translated into Slovenian, back-translated, and subjected to a pilot test. The accepted Slovenian version of the questionnaire was mailed to 2500 randomly selected adult residents of the Republic of Slovenia. Reliability was examined using Cronbach’s alpha for the 1-, 3-, 4-, and 12-factor models addressing health literacy, three main health contexts, four health information processing competencies, and 12 combinations, respectively. Validity was explored with confirmatory factor analysis, univariate analysis, and multiple linear regression.

**Results:**

A total of 517 responses were collected (21% response rate). The highest Cronbach’s alpha was obtained for the 1-factor model (0.950), followed by the 3-, 4-, and 12-factor models. In the confirmatory factor analysis, the 12-factor model provided the most valid results (CFI 0.812; RMSEA 0.067, CI 0.065 to 0.070), followed by the 3-, 4-, and 1-factor models. In the multiple regression model, only the association between self-assessment of health and the health literacy index was statistically significant (*p* < 0.001).

**Conclusion:**

The Slovenian version of HLS-EU-Q47 is a reliable instrument for measuring health literacy. All models of the questionnaire showed reasonable model fit, but none fully satisfied all validity criteria. Respondents differentiated better between the three main health contexts (health care, disease prevention, and health promotion) than the four health information processing competencies (access, understand, appraise, and apply).

**Supplementary Information:**

The online version contains supplementary material available at 10.1007/s11096-023-01610-z.

## Impact statements

- The Slovenian version of HLS-EU-Q47 is a reliable instrument for measuring health literacy.
- A general self-perceived health assessment is a strong predictor of health literacy.
- HLS-EU-Q47 may need to be revised to distinguish more clearly between the four health information processing competencies and to ensure comprehensibility for individuals with varying levels of cognitive and literacy skills.
- In daily practice, pharmacists should be aware of the complexity of information processing and strive to provide advice and services that people can understand and use most effectively with the skills at their disposal.

## Introduction

According to the European Health Literacy Project Consortium (HLS-EU Consortium), health literacy is linked to literacy and entails people’s knowledge, motivation, and competencies to access, understand, appraise, and apply health information to make judgments and take decisions in everyday life concerning healthcare, disease prevention, and health promotion [1]. It is an important and direct determinant of public and individual health, and therefore, limited health literacy is a threat to health care outcomes [2, 3].

Assessing health literacy enables identifying vulnerable populations with low health literacy and undertaking interventions to improve their access to health care, encourage them to play an active role in improving their own health, and ensure health equity [4]. In addition to defining and understanding the meaning of health literacy, it should be comprehensively measured and outcomes across different populations need to be compared. The ideal measurement method should distinguish between functional, interactive, and critical health literacy [5]. Functional health literacy refers to the practical application of literacy skills needed to act effectively in everyday situations. Interactive and critical health literacy refer to more advanced cognitive and literacy skills used in active participation in everyday activities, especially related to health education and communication content. These skills include applying new information to changing circumstances (interactive health literacy) and critically analysing information and using it to gain greater control over life events and situations (critical health literacy). Measurement of health literacy should be standardised in every country to enable designing adequate country-specific measures and to support benchmarking [6].

Several instruments for measuring health literacy have been developed, and one of the most widely used is the European Health Literacy Questionnaire (HLS-EU-Q47) [7]. HLS-EU-Q47 is a self-assessment tool developed by the HLS-EU Consortium, which aimed to design an instrument for collecting data on health literacy in Europe that could provide insights into national perspectives and facilitate comparative analysis of the state of health literacy based on the definition and conceptual model of health literacy, as outlined by Sorensen et al. [1, 7]. The conceptual model distinguishes between three main health contexts of health literacy: health care, disease prevention, and health promotion. It also considers four health information processing competencies: access, understand, appraise, and apply. The four health information processing competencies require specific cognitive and literacy skills such as the ability to seek, find, and obtain health information (access); comprehend the health information (understand); evaluate the health information (appraise); and use the information to make a decision for better health (apply) [1]. In the context of health care, these skills refer to accessing medical information, understanding and evaluating it, and making decisions on medical issues. In the context of disease prevention, these skills refer to accessing information on risk factors for health, understanding and evaluating this information, and making decisions on risk factors for health. In the context of health promotion, these skills refer to learning about health determinants in the social and physical environment, understanding and evaluating this information, and making decisions on health determinants in the social and physical environment. Because HLS-EU-Q47 is a self-assessment questionnaire and therefore has to be completed by the individual, it must be adapted for use in each country to ensure its cultural applicability [8]. Hence, formal national versions of HLS-EU-Q47 are urgently needed [7].

### Aim

The aim of the study was to translate HLS-EU-Q47 into the Slovenian language and to investigate its reliability and validity in Slovenia.

### Ethics approval

The study was approved by the National Medical Ethics Committee of the Republic of Slovenia (registry number 0120-223/2019/4). The cover letter to residents indicated that responses would be collected anonymously and handled in accordance with the General Data Protection Regulation (EU) 2016/679 dated April 27, 2016. The letter also stated that completion of the questionnaire implies acceptance of these conditions and voluntary participation.

## Method

### HLS-EU-Q47 questionnaire

HLS-EU-Q47 measures a person’s ability to manage health-related information, and its conceptual model distinguishes between three main health contexts of health literacy: health care, disease prevention, and health promotion; and four health information processing competencies: access, understand, appraise and apply [1, 7]. These three contexts and the four competencies are used to create different models for understanding health literacy, including a 1-factor model, a 3-factor model, a 4-factor model, and a 12-factor model, as identified by Sørensen et al. (electronic supplementary materials 1, 2, 3, and 4). HLS-EU-Q47 consists of 47 items scored using a 4-level self-assessment scale (very easy, easy, difficult, very difficult).

### Translation and design of the Slovenian version of the HLS-EU-Q47 questionnaire

HLS-EU-Q47 was provided by a research group of the European Health Literacy Project consortium based at Maastricht University in the Netherlands, led by Kristina Sørensen. Dr Jürgen Pelikan, who was part of the questionnaire development group, provided written permission for translation and validation of the questionnaire. The translation process in the current study followed similar procedures described in previous studies [7]. The questionnaire was first sent to two independent translators who translated it from English into Slovenian. The inconsistencies in the translations were reviewed and resolved by the authors, and a third independent translator then translated the questionnaire back into English. This translation was compared with the original document to assess whether the translations matched. During the revision of the translation, special attention was paid to the specifics of the Slovenian language and the cultural background, which the translation was also adapted to. The translation and the original matched in terms of content, such that no additional corrections were required for the Slovenian version. Sociodemographic questions were added to the questionnaire, which contained basic information about the respondents (gender, age, education, statistical region, income, general self-perceived health assessment).

### Pilot test

The full document package, which included a cover letter and the Slovenian version of HLS-EU-Q47 with sociodemographic questions, was pilot tested using an opportunity sample of 10 respondents. Their feedback on the clarity of the documents, the difficulty of completion, and the questionnaire layout, together with additional comments, was collected.

### Study participants and data collection

Residents of the Republic of Slovenia aged 18 years and older were eligible to participate in the study. The required sample size was calculated according to the Nunnally’s rule of thumb of 10 observations per variable [9]. Thus, the minimum number of required responses was 470. Since a response rate of 20–30% was expected, addresses for 2500 random individuals were obtained from the Statistical Office of the Republic of Slovenia. These individuals received the package with the questionnaire in printed form. No reminders were sent, and people choosing to participate returned the completed questionnaires in the enclosed envelopes. Responses were anonymous and were collected from June 2020 to September 2020.

### Statistical analysis

The results of the questionnaires were analysed using IBM SPSS Statistics 27 and IBM SPSS AMOS 27. The answer was reported as missing if it was filled in incorrectly, was not filled in, or if the ‘do not know’ option was selected.

#### Descriptive statistics

Descriptive statistics were used to assess sociodemographic data. Median values are reported with an interquartile range (IQR).

#### Reliability analyses

Internal consistency was tested using Cronbach’s alpha, which indicates the proximity of a group of items and is considered a measure of matrix reliability. A value of 0.70 and above was determined to be an acceptable level of reliability [10]. The reliability of 1-, 3-, 4- and 12-factor models was tested. In addition, testing determined whether any of the items diminished the internal consistency of any factor.

#### Validity analyses

Validity was tested with confirmatory factor analysis (CFA), univariate analysis, and multiple linear regression (MLR). Since the structure of the questionnaire was already known and the research question involved testing a predetermined factor structure, an exploratory factor analysis was not conducted [11, 12]. The CFA was used to determine which model (1-, 3-, 4- or 12-factor model) best fit the data. This analysis was performed using the IBM SPSS AMOS program by creating factor models in relation to the given domains. The data from SPSS with the survey results were imported into AMOS with the missing values which were further replaced with an estimate of the mean values. The fit of the data to the model was examined using the chi-square goodness-of-fit test (χ^2^ test), root mean square error of approximation (RMSEA), comparative fit index (CFI), and chi-square p-value. The values for the χ^2^ test should be less than 3.0 or less than 0.060 for the RMSEA [12]. If the sample size is small and other indices fit the model well, RMSEA values less than 0.080 are acceptable. If the model fits well, the CFI value should be above 0.95; if the model fit is acceptable, the CFI value should be between 0.90 and 0.95; and if the model does not fit, the CFI value would be below 0.90 [11]. Modification indices were not used to improve the fit of a model because there was no strong and defensible theoretical reason for doing so [3, 13]. A p-value of less than 0.05 was considered statistically significant. Univariate analysis was used to explore bivariate correlations. To examine the linear correlations between two or more independent variables and a single dependent variable, MLR was used. The Health Literacy Index was used as the dependent variable and calculated as suggested by the HLS-EU Consortium [8]. The following formula was used: Health Literacy Index = (mean − 1) × (50/3), where mean is the average of all items for each person, 1 is the minimum possible value of the mean, 3 is the range of the mean, and 50 is the selected maximum value of the index. Participants could be classified into four groups: ‘inadequate’ (0–25), ‘problematic’ (> 25–33), ‘sufficient’ (> 33–42), and ‘excellent’ (> 42–50). The independent variables were gender, age, education, region, monthly income, and general self-perceived health assessment. Categorical independent variables with more than two categories were transformed into dummy variables. The forced entry method of regression (SPSS: Enter method) was used. Multicollinearity was examined by the variance inflation factors.

## Results

### Pilot test

The pilot test responses did not identify the need for any further changes to the content of the questionnaire.

### Study participants and sociodemographic characteristics

A total of 517 responses were collected (517/2500; 21% response rate), thus meeting the required sample size. The median age of respondents was 55 years, and 58.2% were female (Table 1). Most of the respondents had secondary education (47.0%) and monthly income less than 700€ (27.5%). Respondents rated their general self-perceived health assessment as good (33.5%), followed by very good (25.7%), acceptable (23.2%), excellent (12.0%), and poor (4.3%).

**Table 1:** Sociodemographic characteristics of participants

Characteristic	Value (n = 517; 100%)
Age in years; median (IQR)	55 (37–66)
Missing	13 (2.5%)
Gender	
Female	301 (58.2%)
Male	215 (41.6%)
Missing	1 (0.2%)
Education	
Primary school or lower education	48 (9.3%)
Secondary school	243 (47.0%)
Diploma	98 (19.0%)
Bachelor’s degree	121 (23.4%)
Missing	7 (1.4%)
Statistical region	
Pomurska	23 (4.4%)
Podravska	78 (15.1%)
Koroška	21 (4.1%)
Savinjska	67 (13.0%)
Zasavska	15 (2.9%)
Posavska	20 (3.9%)
Jugovzhodna	29 (5.6%)
Osrednjeslovenska	128 (24.8%)
Gorenjska	61 (11.8%)
Primorsko-notranjska	20 (3.9%)
Goriška	23 (4.4%)
Obalno-kraška	29 (5.6%)
Missing	3 (0.6%)
Monthly income	
Less than 700€	142 (27.5%)
701–900€	86 (16.6%)
901–1100€	81 (15.7%)
1101–1500€	86 (16.6%)
More than 1500€	56 (10.8%)
Missing	66 (12.8%)
General self-perceived health assessment	
Poor	22 (4.3%)
Acceptable	120 (23.2%)
Good	173 (33.5%)
Very good	133 (25.7%)
Excellent	62 (12.0%)
Missing	7 (1.4%)

IQR, interquartile range; Missing, the answer was completed incorrectly, was not completed, or was answered "don’t know"

### Reliability

The results of the reliability tests of the 1-, 3-, 4-, and 12-factor models are shown in Table 2. With two exceptions, all results had acceptable reliability, meaning that the Cronbach’s alpha was above 0.700. The highest Cronbach’s alpha was obtained for the 1-factor model, followed by the 3-, 4-, and 12-factor models. In the 12-factor model, two factors had particularly low Cronbach’s alpha values and include items 21 and 29. Indices in the 12-factor model that included item 21 were Disease prevention and Understand information, with items 21 *(…understand health warnings about behaviour such as smoking, low physical activity and drinking too much?)*, 22 *(…understand why you need vaccinations?)*, and 23 *(…understand why you need health screenings?).* Item 21 referred to understanding indicators of an unhealthy lifestyle, while the other two items referred to understanding preventive measures (i.e., vaccinations and screenings). The indices in the 12-factor model that included item 29 were Disease prevention and Apply information, with items 29 *(…decide if you should have a flu vaccination?)*, 30 *(…decide how you can protect yourself from illness based on advice from family and friends?)*, and 31 *(…decide how you can protect yourself from illness based on advice from media?).* Item 29 specifically referred to deciding about vaccination, while the other two referred to deciding about general protection against diseases. Omitting items 21 and 29 resulted in higher reliability for the 12-factor model (Cronbach’s alpha above 0.700); however, omitting these items from the 1-, 3-, and 4-factor models decreased the reliability of these models.

**Table 2:** Internal consistency of the Slovenian version of the European Health Literacy Survey Questionnaire

Model	Health-related index	Cronbach’s alpha
1-factor model	HC + DP + HP + A + B + C + D	0.956
3-factor model	HC	0.902
	DP	0.895
	HP	0.904
4-factor model	A	0.886
	B	0.853
	C	0.883
	D	0.815
12-factor model	HC + A	0.772
	HC + B	0.787
	HC + C	0.767
	HC + D	0.754
	DP + A	0.764
	DP + B	**0.678**
	DP + C	0.794
	DP + D	**0.634**
	HP + A	0.825
	HP + B	0.746
	HP + C	0.815
	HP + D	0.820

HC, health care; DP, disease prevention; HP, health promotion; A, access; B, understand; C, appraise; D, apply. Cronbach’s alpha values below 0.70 are highlighted in bold

### Validity

#### CFA

The CFA showed that the 12-factor model provided the highest validity and the 1-factor model provided the lowest with respect to all three indices studied, namely chi-square value, RMSEA, and CFI (Table 3). A closer look at the standardized regression weights for the 12-factor model revealed that item 29 had a low factor loading (< 0.50; electronic supplementary material 5). Since omitting items 21 and 29 resulted in higher reliability of the 12-factor model, the validity of the 12-factor model without these two items was examined and found to be higher than that of the original 12-factor model (χ^2^ value/df 3.242; CFI 0.829; RMSEA 0.066, CI 0.063 to 0.069; *p* < 0.001). The 3-factor model had higher validity than the 4-factor model, suggesting that respondents were better able to differentiate between the three main health contexts than the four health information processing competencies. All models showed reasonable model fit, but none of them fully satisfied the marginal values of all indices. The path diagrams of all factor models from IBM SPSS AMOS are shown in electronic supplementary materials 1, 2, 3, and 4.

**Table 3:** Results of confirmatory factor analysis (standard model) of the Slovenian version of the European Health Literacy Survey Questionnaire

Model	Health-related index	χ^2^ value/df	CFI	RMSEA (CI)	*p* value
1-factor model	HC + DP + HP + A + B + C + D	5.311	0.632	0.091 (0.089–0.094)	< 0.001
3-factor model	HC DP HP	4.466	0.705	0.082 (0.080–0.084)	< 0.001
4-factor model	A B C D	5.292	0.635	0.091 (0.089–0.094)	< 0.001
12-factor model	HC + A + B + C + D DP + A + B + C + D HP + A + B + C + D	3.348	0.812	0.067 (0.065–0.070)	< 0.001

HC, health care; DP, disease prevention; HP, health promotion; A, access; B, understand; C, appraise; D, apply; χ^2^, chi-square; df, degrees of freedom; CFI, comparative fit index; RMSEA, root mean square error of approximation; CI, confidence interval

#### Univariate analysis

Univariate analysis results are reported in Table 4. These results showed that older people were less educated, had lower income, and lower general self-perceived health assessment. Health literacy index decreased with age, and it increased with higher general self-perceived health assessment. Other factors were not significantly correlated with health literacy index.

**Table 4:** Results of univariate analysis of the Slovenian version of the European Health Literacy Survey Questionnaire

	Health literacy index	Gender	Age	Education	Statistical region	Income
Gender	*p* = 0.551 (MW)	–				
Age	***p* = 0.014** (S, r − 0.110)	*p* = 0.187 (MW)	–			
Education	*p* = 0.342 (KW, df 3)	*p* = 0.190 (χ^2^, df 3)	***p* < 0.001** (KW, df 3)	–		
Statistical region	*p* = 0.184 (KW, df 11)	*p* = 0.894 (χ^2^, df 11)	*p* = 0.981 (KW, df 11)	NA	**–**	
Income	*p* = 0.222 (KW, df 4)	***p* = 0.036** (χ^2^, df 4)	***p* = 0.004** (KW, df 4)	***p* < 0.001** (χ^2^, df 12)	NA	–
General self-perceived health assessment	***p* < 0.001** (S, r 0.281)	*p* = 0.772 (MW)	***p* < 0.001** (S, r − 0.460)	***p* < 0.001** (KW, df 3)	*p* = 0.409 (KW, df 11)	***p* < 0.001** (KW, df 4)

HL, KW, Kruskal–Wallis test; MW, Mann–Whitney test; S, Spearman's correlation; r, Spearman's rho; df, degree of freedom; χ^2^, chi-square test; NA, invalid test. Factors significantly correlated are highlighted in bold

#### Multiple linear regression

Regarding health literacy index, participants were classified as ‘inadequate’ (45/517; 8.7%), ‘problematic’ (234/517; 45.3%), ‘sufficient’ (184/517; 35.6%), and ‘excellent’ (54/517; 10.4%). All variance inflation factors were below 2 and the tolerance statistics were all above 0.2, indicating minor multicollinearity among the factors. Only general self-perceived health assessment and age had high proportions on the same small eigenvalue, indicating that the variances of their regression coefficients were dependent. The dependent variable, health literacy index, was positively correlated with general self-perceived health assessment; when the latter increased by one unit on a scale of 1 to 5, the health literacy index increased by 1.910 (*p* < 0.001, Table 5). Lower health literacy index was observed in almost all regions outside the Osrednjeslovenska region, but the result was not statistically significant in most cases. Other independent variables such as gender, age, education attained, and monthly income were not significantly associated with the health literacy index.

**Table 5:** Results of multiple linear regression of the Slovenian version of the European Health Literacy Survey Questionnaire

Sociodemographic characteristics	Unstandardized coefficients		Standardized coefficients	t value	*p* value
	B	Std. Error	Beta		
Constant	27.729	1.904		14.565	**< 0.001**
Female versus male	0.598	0.640	0.045	0.934	0.351
Age	0.005	0.019	0.015	0.277	0.782
Education					
Primary school or lower education versus Secondary school	1.359	1.123	0.062	1.210	0.227
Diploma versus Secondary school	− 1.327	0.889	− 0.079	− 1.493	0.136
Bachelor’s degree versus Secondary school	− 0.230	0.939	− 0.015	− 0.244	0.807
Statistical region*					
Jugovzhodna versus Osrednjeslovenska	− 3.702	1.400	− 0.132	− 2.645	**0.008**
Income					
701–900€ versus less than 700€	− 0.743	0.910	− 0.045	− 0.817	0.414
901–1100€ versus less than 700€	0.588	0.960	0.035	0.612	0.541
1101–1500€ versus less than 700€	0.893	1.009	0.054	0.885	0.377
More than 1500€ versus less than 700€	− 0.863	1.263	− 0.044	− 0.683	0.495
General self-perceived health assessment	1.910	0.347	0.305	5.497	**< 0.001**
*Model summary*					
R Square	0.123				
Adjusted R Square	0.079				

*Statistical regions not significantly correlated with the health literacy index are not shown in the table

Factors significantly correlated are highlighted in bold

## Discussion

### Statement of key findings

In this study of 517 respondents, the Slovenian version of HLS-EU-Q47 proved to be a reliable instrument for measuring health literacy in the adult population in Slovenia. With regard to validity, the 12-factor model had the highest model fit, but none of the questionnaire models fully met all validity criteria. The higher validity of the 3-factor model compared with the 4-factor model implies that the participants perceived the three main health contexts (health care, disease prevention, health promotion) as distinct factors to a greater extent than they differentiated the four health information processing competencies (access, understand, appraise, apply).

### Strengths and weaknesses

To our knowledge, this study is the first translation and validation of HLS-EU-Q47 in Slovenia. Furthermore, a comprehensive investigation of reliability and validity was conducted, which can serve as a basis for further development of standardized instruments to measure health literacy in different countries. Nevertheless, HLS-EU-Q47 is a perception-based questionnaire, so respondents may underestimate or overestimate their abilities assessed in the questionnaire. Therefore, measuring health literacy with a performance-based instrument would be useful to confirm our findings. The health literacy index could be further overestimated if completed questionnaires were returned mainly by participants who considered themselves more health literate. Another potential limitation of this study is the low response rate and a higher proportion of female respondents than male, which could potentially affect the generalizability of the results. Further, additional information on respondent characteristics could be collected to potentially identify other factors that influence health literacy or arise from low health literacy, such as certain diseases, body mass index, alcohol consumption, smoking, use of health services, medication adherence and others [14, 15].

### Interpretation and further research

#### Reliability

We obtained high values for Cronbach’s alpha for all models in the internal consistency analysis, indicating that the Slovenian version of HLS-EU-Q47 is a reliable instrument. We looked more closely at the 12-factor model because only items 21 and 29 had unacceptable reliability. When these items were eliminated, Cronbach’s alpha for all dimensions reached an acceptable level of reliability. A low alpha value could be due to a small number of questions or poor correlation between items or heterogeneous constructs [16]. Therefore, our results are probably explained by the different number of items included in the models as the 1-factor model initially included all 47 items, whereas the other models included fewer items. However, the higher reliability of the 12-factor model without items 21 and 29 may be due to the lower correlation of these two items to each of the indices of the 12-factor model. Similar studies using HLS-EU-Q47 have also determined an acceptable level of reliability with Cronbach’s alpha 0.900 or higher. These studies were conducted in different countries [17–21] and even with different groups within a population, for example, patients with breast cancer [22] or type 2 diabetes [23].

#### Validity

The model that best fit our data according to CFA was the 12-factor model. Based on the internal consistency results and the CFA results after items 21 and 29 were omitted from the 12-factor model, we suggest that these two items be deleted from the questionnaire. Similar results were observed in the study by Huang et al. [22] in which women with breast cancer completed HLS-EU-Q47 and deletion of item 29 resulted in an adequate fit of the data. The authors concluded that this finding could have been due to differential acceptance of influenza vaccination, which is influenced more by government health policies than by participants’ health literacy.

None of the models in our study fully satisfied the marginal values for all indices of the CFA. The reason may be that the structure of the original questionnaire was determined in advance by the definition of health literacy and not by the questionnaire responses. Interestingly, the 3-factor model had higher validity than the 4-factor model, suggesting that distinguishing between the four health information processing competencies of HLS-EU-Q47 likely requires more advanced literacy skills than distinguishing between the three main health contexts. We assume that the distinction between the meanings of the words "access," "understand," "appraise," and "apply" presupposes a high level of literacy, which could explain why respondents mainly connected questions with the same content and did not recognize the difference related to the difficulty of the skill explained by these specific words. Other studies also had difficulty confirming the latent structure of HLS-EU-Q47 and noted the risk of local dependence [22, 23], implying that the wording of the items may have been too similar to permit perceiving differences in the required abilities. Therefore, Huang et al. suggested reformulating the items to improve discrimination between them [22], and Finbraten et al. recommended developing a unidimensional measurement scale based on HLS-EU-Q47 [23].

According to the univariate analysis, older and less educated social groups with low income in Slovenia have a higher proportion of people with low health literacy. However, the multivariate model did not find any association between the health literacy index and sociodemographic characteristics, indicating that other factors may be more influential. According to the univariate analysis and multivariate linear regression model, the strongest predictor of health literacy in our study was general self-perceived health assessment. The HLS-EU survey, which compared health literacy across European countries, identified the existence of a social gradient in health literacy and reported that low health literacy was associated with financial deprivation, low social status, low education, or old age, as confirmed by a raw bivariate correlations and a multivariate linear regression model [14]. Similarly, a study conducted in Slovenia found that age, but not education, was a predictor of medication literacy [24]. Taken together, the results of our study and others suggest that while sociodemographic factors may play a role in health literacy, other factors may also be important and more research is needed to understand them better. HLS-EU-Q47 refers to self-perceived measurement and reflects individual perception and competence, so this should be considered when interpreting results as well.

Further research should focus specifically on the highlighted challenges in confirming the validity of other translated versions of HLS-EU-Q47. Nonetheless, other national versions of questionnaires have previously been confirmed as valid [21, 25, 26], and we also observed an increase in validity for models with more factors, suggesting that the 12-factor model construct is the most appropriate. Omitting some items, especially 21 and 29, could be considered to achieve a good fit of the models. We believe that the Slovenian version of HLS-EU-Q47 has the potential to measure health literacy and identify the specific population groups with low health literacy. It can serve as a useful tool for planning concrete interventions to improve specific aspects of health literacy in these populations and allow comparisons across countries.

## Conclusion

The Slovenian version of HLS-EU-Q47 is a reliable instrument for measuring health literacy in the adult population in Slovenia. None of the models fully satisfied all validity criteria. The 3-factor model had higher validity than the 4-factor model; therefore, distinguishing between the four health information processing competencies of HLS-EU-Q47 likely requires more advanced literacy skills than distinguishing between the three main health contexts.

### Supplementary Information

Below is the link to the electronic supplementary material.

Electronic supplementary material 1

Electronic supplementary material 2

Electronic supplementary material 3

Electronic supplementary material 4

Electronic supplementary material 5
