# Supplementary material for: Translation and validation of the European Health Literacy Survey Questionnaire (HLS-EU-Q47) into the Slovenian language
Source: Int J Clin Pharm. 2023 Jun 24;45(6):1387–95. doi: 10.1007/s11096-023-01610-z (PMC10682039; doi:10.1007/s11096-023-01610-z)
Supplement: Supplementary file 2 — Electronic supplementary material 2 [file 11096_2023_1610_MOESM2_ESM.docx]

**Translation and validation of the European Health Literacy Survey Questionnaire (HLS-EU-Q47) into the Slovenian language**

Nuša Japelj ^1^, Nejc Horvat ^1^

^1^ University of Ljubljana, Faculty of Pharmacy, Department of Social Pharmacy, Askerceva cesta 7, 1000 Ljubljana, Slovenia

**Correspondence to** Nejc Horvat, nejc.horvat@ffa.uni‑lj.si


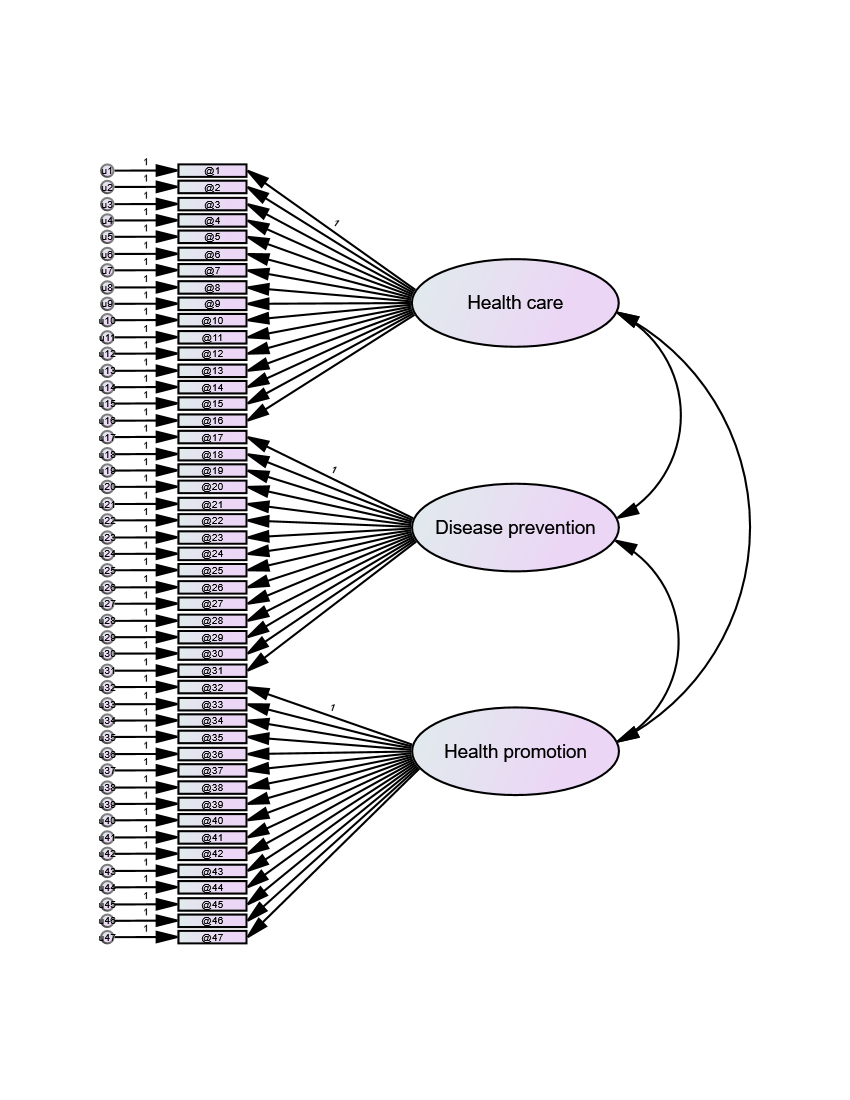


Figure 2 The path diagrams of a 3-factor model from IBM SPSS AMOS, which addresses three main health contexts of health literacy: health care, disease prevention, health promotion.
