# Supplementary material for: Translation and validation of the European Health Literacy Survey Questionnaire (HLS-EU-Q47) into the Slovenian language
Source: Int J Clin Pharm. 2023 Jun 24;45(6):1387–95. doi: 10.1007/s11096-023-01610-z (PMC10682039; doi:10.1007/s11096-023-01610-z)
Supplement: Supplementary file 3 — Electronic supplementary material 3 [file 11096_2023_1610_MOESM3_ESM.docx]

**Translation and validation of the European Health Literacy Survey Questionnaire (HLS-EU-Q47) into the Slovenian language**

Nuša Japelj ^1^, Nejc Horvat ^1^

^1^ University of Ljubljana, Faculty of Pharmacy, Department of Social Pharmacy, Askerceva cesta 7, 1000 Ljubljana, Slovenia

**Correspondence to** Nejc Horvat, nejc.horvat@ffa.uni‑lj.si


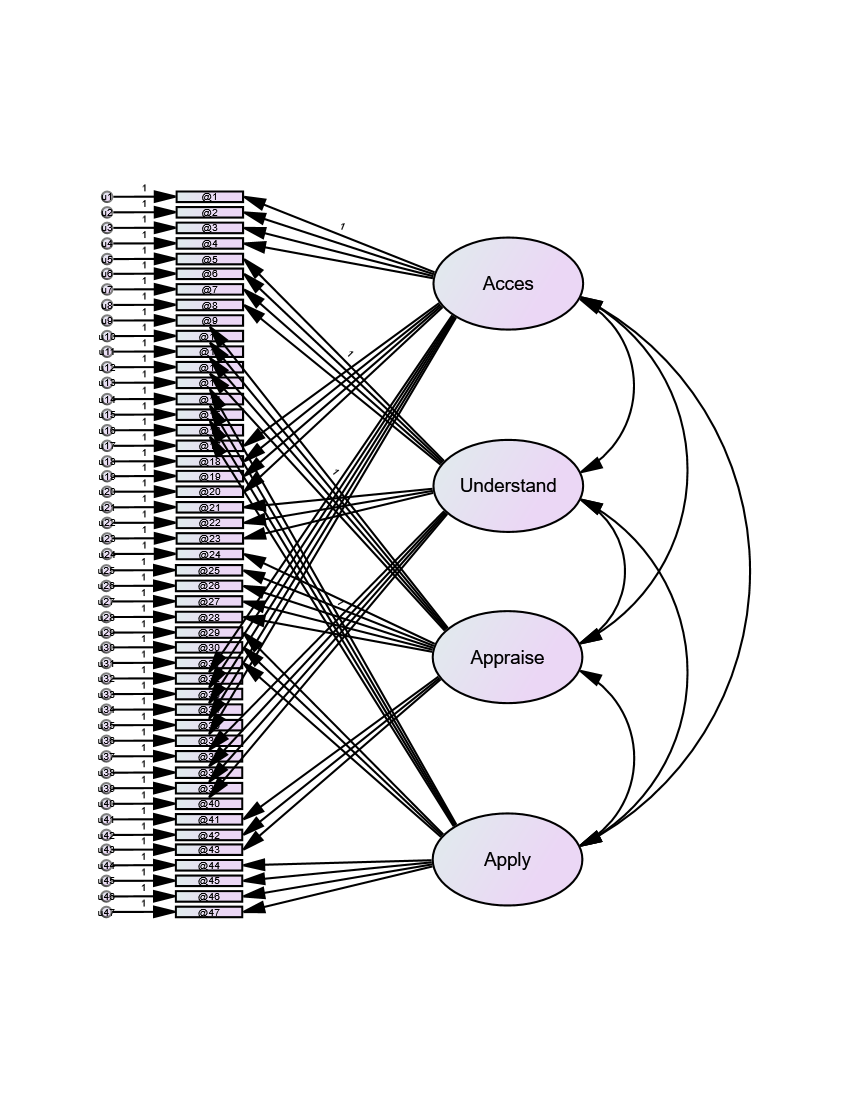


Figure 3 The path diagrams of a 4-factor model from IBM SPSS AMOS, which addresses four health information processing competencies: access, understand, appraise, apply.
