# Supplementary material for: Translation and validation of the European Health Literacy Survey Questionnaire (HLS-EU-Q47) into the Slovenian language
Source: Int J Clin Pharm. 2023 Jun 24;45(6):1387–95. doi: 10.1007/s11096-023-01610-z (PMC10682039; doi:10.1007/s11096-023-01610-z)
Supplement: Supplementary file 4 — Electronic supplementary material 4 [file 11096_2023_1610_MOESM4_ESM.docx]

**Translation and validation of the European Health Literacy Survey Questionnaire (HLS-EU-Q47) into the Slovenian language**

Nuša Japelj ^1^, Nejc Horvat ^1^

^1^ University of Ljubljana, Faculty of Pharmacy, Department of Social Pharmacy, Askerceva cesta 7, 1000 Ljubljana, Slovenia

**Correspondence to** Nejc Horvat, nejc.horvat@ffa.uni‑lj.si


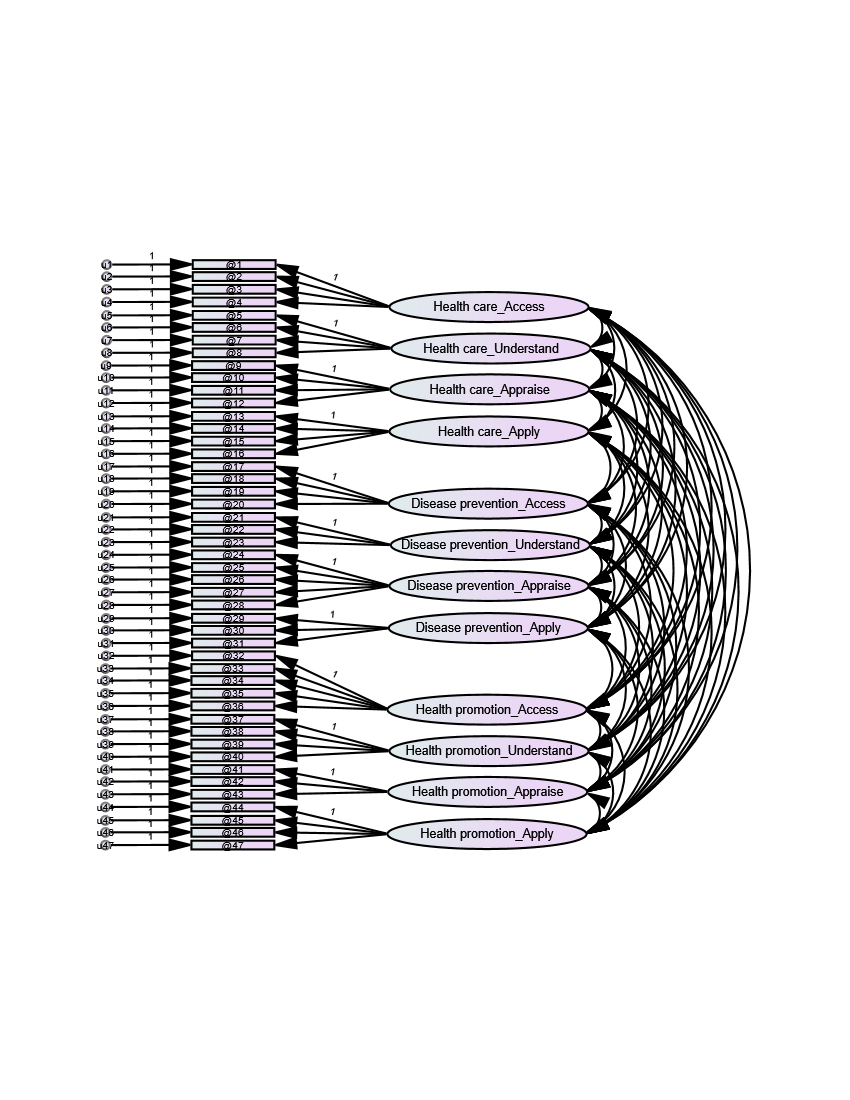


Figure 4 The path diagrams of a 12-factor model from IBM SPSS AMOS, which addresses 12 combinations of three main health contexts (health care, disease prevention, health promotion) and four health information processing competencies (access, understand, appraise, apply).
